# Parental views on acute otitis media (AOM) and its therapy in children - results of an exploratory survey in German childcare facilities

**DOI:** 10.1186/s12887-015-0516-3

**Published:** 2015-12-01

**Authors:** Sibylle Kautz-Freimuth, Marcus Redaèlli, Christina Samel, Daniele Civello, Sibel V. Altin, Stephanie Stock

**Affiliations:** Institute of Health Economics and Clinical Epidemiology, University Hospital of Cologne (AöR), Gleueler Straße 176-178, 50935 Cologne, Germany; Institute of General Practice, Medical Faculty, Heinrich-Heine-University Düsseldorf, Moorenstraße 5, 40225 Düsseldorf, Germany

**Keywords:** Acute otitis media in children, Antibiotic treatment, Exploratory survey, Pediatrics, Health service research, Parental views

## Abstract

**Background:**

Acute otitis media (AOM) is one of the main reasons for medical consultation and antibiotic use during childhood. Although 80 % of AOM cases are self-limiting, antibiotic prescription is still high, either for physician- or for parent-related factors. This study aims to identify parental knowledge about, beliefs and attitudes towards, and experiences with AOM and its therapy and thus to gain insights into parents’ perspectives within the German health care system.

**Methods:**

An exploratory survey was conducted among German-speaking parents of children aged 2 to 7 years who sent their children to a childcare facility. Childcare facilities were recruited by convenience sampling in different urban and rural sites in Germany, and all parents with children at those facilities were invited to participate. Data were evaluated using descriptive statistical analyses.

**Results:**

One-hundred-thirty-eight parents participated. Of those, 75.4 % (*n* = 104) were AOM-experienced and 75.4 % (*n* = 104) had two or more children. Sixty-six percent generally agree that bacteria cause AOM. 20.2 % generally agree that viruses cause AOM. 30.5 % do not generally agree that viruses cause AOM. Eight percent generally agree that AOM resolves spontaneously, whereas 53.6 % do not generally agree. 92.5 % generally (45.7 %) and partly (42.8 %) agree that AOM needs antibiotic treatment. With respect to antibiotic effects, 56.6 % generally agree that antibiotics rapidly relieve earache. 60.1 % generally agree that antibiotics affect the gastrointestinal tract and 77.5 % generally agree that antibiotics possibly become ineffective after frequent use. About 40 % generally support and about 40 % generally reject a “wait-and-see” strategy for AOM treatment. Parental-reported experiences reveal that antibiotics are by far more often prescribed (70.2 %) than actively requested by parents (26.9 %).

**Conclusions:**

Parental views on AOM, its therapy, and antibiotic effects reveal uncertainties especially with respect to causes, the natural course of the disease and antibiotic effects on AOM. These results indicate that more evidence-based information is needed if parents’ health literacy in the treatment of children with AOM is to be enhanced. The discrepancy between reported parental requests for antibiotics and reported actual prescriptions contradicts the hypothesis of high parental influence on antibiotic use in AOM.

**Electronic supplementary material:**

The online version of this article (doi:10.1186/s12887-015-0516-3) contains supplementary material, which is available to authorized users.

## Background

Acute otitis media (AOM) is one of the most common infectious diseases in young children and represents one of the main reasons for doctor consultations during childhood [1]. In addition AOM is the leading cause of antibiotic treatment in children [2–4]. According to data from the USA, around 50 to 85 % of all children up to the age of three have had at least one episode of AOM [5, 6] and about 40 % have experienced six or more episodes by the age of seven [6]. According to the German Health Interview and Examination Survey for Children and Adolescents (KIGGS) the 12-month prevalence of AOM across all age groups is 11 % and rises to 22.9 % between the ages of 3 and 6 years [7]. AOM can be caused by bacteria or viruses as well as by mixed pathophysiological mechanisms [8–12]. The bacterial pathogens mostly involved in AOM development are Streptococcus pneumonia, Haemophilus influenza, and Moraxella catarrhalis [13], but it has been suggested that the spectrum of predominant bacterial pathogens responsible for AOM might change due to previous antibiotic prescriptions [13] or to pneumococcal vaccination [14, 15]. AOM episodes typically occur subsequent to a viral upper respiratory infection, but the underlying mechanisms for the interaction between the different pathogens are still being investigated [9]. Despite the frequent involvement of bacteria in the pathogenesis of AOM, an antibiotic treatment is not imperative, with research showing that 80 % of uncomplicated AOM cases in children resolve spontaneously within 48 to 72 h without antibiotic therapy [16]. Nevertheless, data from the USA and other countries indicate that up to 80 % of medical consultations due to otitis media in children still result in an antibiotic prescription [4, 17, 18].

In the light of the rising prevalence of antibiotic resistance in bacteria [19], the arguments in favour of reducing antibiotic overuse/misuse are compelling. One decisive approach is to avoid antibiotics if not indicated or not superior to symptomatic treatment. A Cochrane Review has recently demonstrated that immediate antibiotic therapy in children with AOM is not superior to an observational therapy (“wait-and-see”) [20]; a therapy where children receive symptomatic analgesic treatment and an antibiotic is not given unless symptoms fail to improve within 48 to 72 h after onset [21]. Currently there is a broad consensus that antibiotics are most beneficial in children younger than 2 years of age with bilateral AOM, and in children with both acute otitis media and otorrhoea [20, 22]. For most other children with mild unilateral AOM, an observational approach seems justified [22–25]. Despite this existing evidence for this strategy the proportion of antibiotic treatment in children with AOM is still high [26]. Possible reasons for physicians over-prescribing antibiotics include physician inertia, lack of detailed knowledge, insufficient use of appropriate analgesia or uncertain diagnosis [27]. With respect to parental influence on the prescription of antibiotics, there is evidence that parental perspectives can indeed have a marked influence on therapeutic decisions [28, 29], and for AOM a certain proportion of parents actively demand antibiotics [30]. Additionally, perceived parental expectations have been identified as one determinant in antibiotic prescription through pediatricians [29, 31]. Prior studies indicate that parental socio-demographic factors, such as educational level, age, or having more than one child can affect parental knowledge and attitudes towards AOM and its therapy [30, 32] and thus might also be relevant. It has been shown that the use of shared decision-making (SDM) in medical consultation is highly influenced by the parental health literacy level, indicating that limited health literacy facilitates a patriarchal relationship between physicians and parents and increases the tendency to follow physician recommendations [33]. Moreover, there is evidence that SDM may lead to less antibiotic prescription and higher levels of parental satisfaction in the treatment of AOM [34]. Therefore, supporting parent-physician interaction and promoting understanding between parents and physicians seems a promising approach to enhancing rational antibiotic prescription in children with AOM.

The aim of this study is to identify parental knowledge, beliefs, attitudes, and experiences with regard to AOM and its therapy and thus to gain initial insights into parental perspectives within the German health care system and to provide a better understanding of non-medical determinants of therapeutic decisions, which may help to enhance SDM in the treatment of children with AOM. We hypothesize that parental knowledge with regard to causes of AOM, best treatment of earache and effects of antibiotics is fairly limited. We further hypothesize that the high use of antibiotic in children with AOM is due to parental preference for antibiotics rather than for non-antibiotic options or a “wait-and-see” strategy. Finally, we hypothesize that parent-related factors such as previous AOM-experience or socio-demographics, do have an impact on decisions related to AOM therapy. By analyzing these research questions, we aim to contribute valuable insights to the ongoing discussion in health services research on whether the parents or the health care professionals are the ones preferring or demanding specific treatment options, especially the use of antibiotics.

## Methods

### Study design, participants and setting

An exploratory survey among German-speaking parents of children aged 2 to 7 years was conducted between January and October 2013. To reach this target group, we recruited childcare facilities by convenience sampling at different sites in the western part of Germany aiming to involve facilities with different pedagogical concepts and thus address a wider spectrum of parent types. The childcare facilities were located in seven towns with population sizes ranging from cities over a million inhabitants to medium-sized towns with 20,000 to 100,000 inhabitants. Five childcare facilities were situated in more rural settings and ten in urban settings. All parents connected to the addressed childcare facilities were invited to participate in the survey. A questionnaire with a cover letter explaining the study objective was distributed to each parent’s pigeonhole in the childcare facility. Only one parent per family was supposed to fill in the questionnaire, even if several children from that family attended the same childcare facility. Parents were asked to return the questionnaire anonymously in a sealed box, which was not opened until data capture and analysis commenced. All childcare facilities had given consent to their participation and distributed questionnaires to the parents. In the specified time period 15 childcare facilities could be recruited, and overall, 710 parents were addressed. As there were no comparable studies available that examined our research questions in a German sample, a middle-sized effect for calculating correlations was assumed, which lead to a minimal number of 107 parents to be questioned [35, 36]. By the end of the recruiting time a sample size of 138 parents representing a total of 278 children was achieved.

### Questionnaire development

To determine the content and structure of the questionnaire a qualitative approach was chosen derived from a concept that was developed by Jónsson and Haraldsson in 2002 [37]. According to their “parents’ explanatory model” there are three issues that mainly influence parents’ perspectives on AOM in their children: (1) perceptions on the causes of AOM, (2) ideas on disease threats due to AOM, and (3) attitudes towards the treatment of AOM (e.g. use of antibiotics). Additionally, we expanded the domains by conducting a literature search to identify AOM-related factors that account for parental knowledge/beliefs, attitudes, and experiences in more detail. To develop the questionnaire, we used these domains as a structural and textual basis and derived an expanded model by discussing the structure of the questionnaire in an expert group consisting of physicians, nurses, health economists, and parents. The aim of the brainstorming was to identify and contextualize topics related to parental knowledge, beliefs, attitudes and experiences related to AOM in children. Eligible domains were then operationalized in a structured discussion process between the experts.

The questionnaire was then revised based on the discussion results and pilot-tested in a two-phase pretest to verify its clarity, comprehensibility and practicability. Testing was performed using the concurrent-think-aloud-method with six participants [38–40] followed by standard pretests with eight volunteers [41]. The final content structure of the questionnaire is presented in Table 1.

**Table 1:** AOM-related topics used in the questionnaire

Parental knowledge/beliefs about AOM	
• Causes of AOM • Symptoms of AOM • Course of the disease • Treatment options • Effects of antibiotics	
Parental attitudes towards AOM treatment	
• Importance of contact partners • Relevant media for obtaining information • Attitudes towards use of antibiotic in own child	
Parental experiences with AOM treatment	
• Frequency of AOM episodes • Choice of health care practitioner • Parental requests for treatment • Actual doctor’s prescription	

The questionnaire consisted of 15 domains with a total of 53 items. A translated English version is accessible in Additional file 1. Each domain consisted of one question with between one and seven corresponding items. Eleven of the domains, with a total of 47 items, referred to aspects of knowledge, attitudes and experiences. They were formulated as closed questions. Eight of them used a five-point Likert rating scale [42], the three remaining domains used categorical scales. The five-point Likert scale (levels of agreement: from “fully agree” to “don’t agree at all”) was chosen to allow for gradual classification of respondents’ opinions [43, 44]. All items concerning knowledge had the additional answer option “don’t know”. Two domains referring to respondents’ experiences used a five-point Likert scale for frequency (from “always” to “never”). We calculated the descriptive analyses by building the following categories: “generally agree”, “partly agree”, “do not generally agree” or “always/often”, “sometimes”, “rarely/never”. Four domains used categorical scales to record answers on socio-demographic data. Two domains addressed the current age of the respondents and their children by asking the respondents to record the respective ages. Multiple answers were not permitted in any domain.

### Data analysis

The questionnaires were scanned and then processed using Remark Office OMR™ Version 8. A random portion of 20 % of the questionnaires was manually checked for scanning errors. The data were then transferred to IBM SPSS Statistics version 21 for statistical analysis. Descriptives were performed using frequencies and counts, contingency tables were evaluated using Fisher’s exact test. Since prior studies indicate that there are socio-demographic determinants of parental AOM knowledge and attitudes, correlations were calculated using–where applicable–Pearson’s correlation coefficient, and for categorical data Spearman’s rho. To adjust for multiple testing, the Bonferroni-Holm method was applied to control for the familywise error rate.

As a guiding measure for overall parental experience on children’s health the group of parents with two or more children (“several-child parents”) was compared to the group of parents with one child (“single-child parents”). The hypothesis that “several-child parents” are more experienced in children’s health is supported in a study by Aku-Baker et al., who reported a significant correlation between the number of children and the mothers’ knowledge and ability to cope with fever in their child [45].

### Ethical considerations

The study was a survey involving questionnaires. Participation was voluntary and anonymous collection and data analysis was guaranteed through anonymous questioning, questionnaire collection, and analysis. All participants gave consent for the results to be published. In a pre-study consultation with the ethic committee of the University Hospital of Cologne, we were advised that an approval through the ethics committee was not necessary.

## Results

### Distribution of questionnaires and response rate

The complete number of distributed questionnaires (*n* = 710) corresponded exactly to the number of families with at least one child in the participating childcare facilities. In total, 138 questionnaires were returned, which results in a response rate of 19.4 %. These questionnaires were included in the data analysis.

### Socio-demographic data

Table 2 summarizes the socio-demographic characteristics of the respondents (n = 138) and their children (*n* = 278). The majority of children (70.14 %) were aged between 2 and 7 years representing the main age category of interest for our survey. All but two respondents had at least one child within this age span.

**Table 2:** Demographic characteristics of the respondents (*n* = 138) and their children (*n* = 278)

Respondents	*N* (138)	Percent
Age (years)		
Mean ± SD	38.13 ± 10.78	
Median	38	
Range	26–49	
Gender		
Female	131	94.9
Male	7	5.1
Education (degree)		
Middle school certification	4	2.9
Intermediate high school certification	23	16.7
Final high school certification	37	26.8
University degree	73	52.9
Living environment		
Urban	86	62.3
Rural	50	36.2
Single parent		
Yes	7	5.1
No	125	90.6
Number of children (per parent)		
1 child	34	24.6
2 children	72	52.2
3 children	28	20.3
4 children	4	2.9
Experience with AOM in own child		
Yes	104	75.4
No	33	23.9
Respondents with AOM experience	*N* (104)	%
Number of AOM episodes		
< 3 times	43	41.4
3–10 times	51	49.0
> 10 times	10	9.6
Health service utilization in AOM		
Pediatrician	63	60.6
General practitioner	3	2.9
ENT specialist	7	6.7
First aid pediatrician service	4	3.8
Emergency service in hospital	3	2.9
Not answered	24	23.1
Children (of all respondents)	*N* (278)	%
Children’s age (years, *n* = 278)		
0–1 year	27	9.7
2–7 years	195	70.1
8–17 years	53	19.1
18 years and older	3	1.1
Mean ± SD (years)	5.37 ± 3.55	
Median (years)	5	
Children’s health insurance (*n* = 278)		
Statutory	187	67.3
Private	81	29.1

### Parental knowledge/beliefs about AOM, treatment of earache and effects of antibiotics

**Fig. 1:**
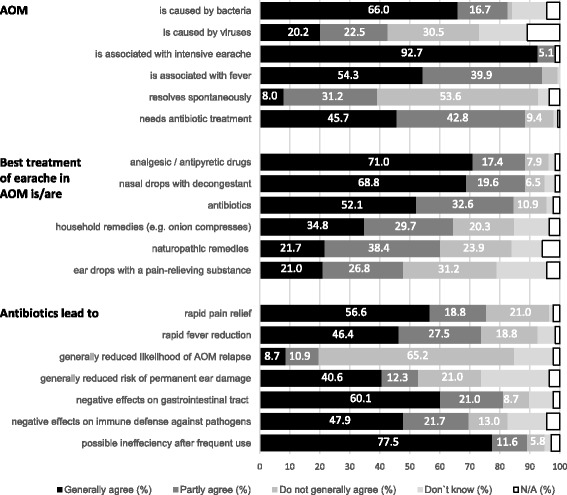
Parental knowledge/beliefs about AOM, best treatment of earache in AOM, and effects of antibiotics (*n* = 138); Generally agree = fully/mostly agree; Do not generally agree = don’t really agree/don’t agree at all; N/A = No answer

Aspects of knowledge and beliefs on AOM, treatment of earache in AOM, and effects of antibiotics in AOM were surveyed using 19 items. The results are presented in Fig. 1. For the cause of AOM, 66 % of all respondents generally agree that bacteria cause AOM. 20.2 % generally agree that viruses cause AOM. 30.5 % do not generally agree that viruses cause AOM. A relatively high proportion of respondents states not to knowing the cause (11.6 % for bacteria, 15.9 % for viruses), and 4.3 and 10.9 %, respectively, do not answer. The view that AOM is caused by viruses meets significantly less approval from parents with increasing age (*p* < 0.05), with AOM experience (*p* < 0.05), who live in an urban environment (*p* < 0.05), and who are single parents (*p* < 0.05). Concerning symptoms, 92.7 % of the parents generally agree that intensive earache is associated with AOM, and 53.4 % generally agree that fever is part of AOM. With respect to the course of the disease, 8 % generally agree that AOM resolves spontaneously, whereas 53.6 % do not generally agree. This view is confirmed by 92.5 % of the respondents, who generally (45.7 %) and partly (42.8 %) agree that AOM needs antibiotic treatment.

Most of the parents consider analgesic/antipyretic drugs (71 %) and nasal drops with decongestant (68.8 %) as being the best treatment for earache (generally agree), but 52.1 % view antibiotics as being the best therapy (generally agree). There is no clear preference for other treatments (household remedies, naturopathic remedies, eardrops), and a relatively high proportion of parents do not know (11.6, 10.2, 16.7 %, respectively). 3.6, 5.8, 4.3 %, respectively, refuse to answer. With regard to antibiotic effects, 56.6 % generally agree that antibiotics lead to rapid pain relief and 46.6 % generally agree that they lead to rapid fever reduction. 8.7 % generally agree that they reduce AOM relapse, whereas 65.2 % do not generally agree. A risk-reducing effect on permanent ear damage is generally affirmed by 40.6 and 26.1 % do not know or do not answer. Concerning undesired effects, the majority generally agrees that antibiotics affect the gastrointestinal tract (60.1 %) and possibly become ineffective after frequent use (77.5 %). Parents holding a university degree believe significantly less often than those without a university degree that antibiotics negatively affect the gastrointestinal tract (*p* < 0.05) and become inefficient after frequent use (*p* < 0.05).

### Parental attitudes towards AOM treatment of their own child

Three domains consisting of 15 items asked for parental attitudes concerning the treatment of AOM. On the topic of the relevance of contact partners, 89.9 % of the respondents see the pediatrician’s opinion as being of great importance (generally agree), whereas 37.7 % rate the family doctor’s opinion being greatly important (generally agree). Close relatives, other parents, teachers in child-care centers and friends who are health care professionals are mainly rated as being of partly, little or no importance. Among the sources of information, the internet takes the first place (46.4 % generally very helpful), followed by books (33.3 % generally very helpful). Radio, television, newspapers and magazines are of little importance.

One domain involving five items addresses parental attitudes towards their willingness to follow a “wait-and-see” strategy in their child with AOM (Table 3). Almost 40 % of the respondents generally agree with not using antibiotics until symptoms persist for 2 days (39.1 %) or worsen overnight (38.4 %). Around the same proportion does not generally agree to delay antibiotic therapy for 2 days (43.5 %) or in case of severe symptoms (44.2 %). In the latter case, “several-child parents” would rather give an antibiotic compared to “single-child parents” (*p* < 0.01). In contrast, 32.6 % of parents generally agree to wait even if the child severely suffers. Previous AOM-experience does not affect this attitude; neither do any of the demographic factors (age, education, living environment).

**Table 3:** Parental willingness to follow a “wait-and-see” strategy in their child with AOM (*n* = 138)

Willingness to wait^a^	Level of agreement (%)			
	Generally agree	Partly agree	Do not generally agree	No answer
Yes; use of antibiotics only when symptoms persist for 2 days	39.1	12.3	43.5	5.1
Yes; use of antibiotics only when symptoms worsen overnight	38.4	23.2	33.3	5.1
Yes; consult the doctor again when symptoms worsen	27.6	13	55.1	4.3
No; immediate use of antibiotics when child experiences severe symptoms	44.2	18.9	32.6	4.3
No; immediate use of antibiotics out of concern about AOM-worsening	25.4	22.5	47.8	4.3

Generally agree = fully/mostly agree

Do not generally agree = don’t really agree/don’t agree at all

^a^For better clarity the patterns of behavior are presented in a shortened version

### Parental experiences with AOM treatment

Four domains including 12 items refer to parental experiences with respect to medical treatment of AOM. This part of the survey only includes respondents who had experienced at least one episode of AOM in their child (*n* = 104, 75.4 %). Forty-three parents have seen less than three episodes, 61 three or more. In this situation, 60.6 % consulted their pediatrician. 23.1 % (*n* = 24) gave no answer.

**Fig. 2:**
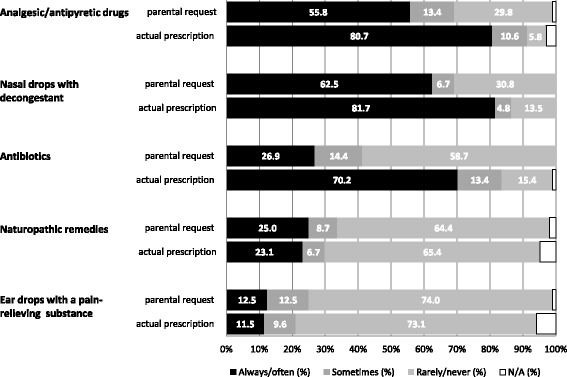
Percentages of parental requests for therapeutic agents for their child with AOM and reported actual prescriptions (*n* = 104); N/A = No answer

Two domains address the questions as to what kind of medical therapy parents wished to have prescribed for their child with AOM and what therapy was actually prescribed by the doctor (Fig. 2). Most often parents ask for nasal drops with decongestant (62.5 %) and analgesic/antipyretic drugs (55.8 %). Corresponding actual prescriptions are 81.7 % for nasal drops and 80.7 % for analgesic/antipyretic drugs, which turns out to be the same tendency but to a higher extent compared to parental requests. Parental requests for naturopathic drugs and eardrops with a pain-relieving substance are relatively rare and the same tendencies are found with respect to the corresponding actual prescriptions.

Comparing the rates of parental requests and actual prescription of antibiotics, we find a striking discrepancy: While only 26.9 % of the parents state having always/often asked for an antibiotic, 70.2 % state that their child was always/often prescribed one. On the other hand, 58.7 % report having rarely/never asked for an antibiotic, while only 15.4 % state having rarely/never received a prescription. 96.4 % of the parents who have always/often asked for an antibiotic (*n* = 28), always/often received a prescription for one, whereas 65 % of the parents who rarely/never asked for an antibiotic (*n* = 60) always/often received a prescription for one.

We evaluated possible differences concerning knowledge and attitudes between parents who wished for an antibiotic and those who did not by comparing the answer categories “always/often/sometimes/rarely” (*n* = 63) to the category “never” (*n* = 41): Compared to parents who never asked for an antibiotic, parents who did wish for one for their child agree more often that antibiotics are the best therapy for earache in AOM (*p* < 0.05) and lead to rapid pain relief (*p* < 0.05). In addition, they disagree more often with the statement that antibiotics negatively affect the infantile gastrointestinal flora (*p* < 0.05) and have less faith in household remedies for treating earache (*p* < 0.05). Knowledge concerning cause, symptoms and course of the disease does not differ between the two groups.

### Impact of parental experience of AOM and children’s health

To assess the impact of experience with AOM on parental knowledge and attitudes the answers of AOM-experienced parents (*n* = 104) were compared to those of non-AOM experienced parents (*n* = 34). Only one in a total of 34 related items in this comparison differs significantly: Compared to non-AOM-experienced parents, AOM-experienced parents agree less often with the statement that AOM is caused by viruses (*p* < 0.05).

To investigate the influence of the number of children per family on the parental responses concerning knowledge/beliefs, attitudes, and experiences the group of “several-child parents” (*n* = 104) was compared to the group of “single-child parents” (*n* = 34). Compared to “single-child parents”, “several-child parents” significantly more often consider bacteria to cause AOM (*p* < 0.05) and nasal drops with decongestant (*p* < 0.05) and naturopathic remedies (*p* < 0.05) to be the best treatment for earache. However, they agree significantly less often that AOM is associated with fever (*p* < 0.05) and that antibiotics are the best therapy for earache (*p* < 0.05). For “several-child parents” close relatives (*p* < 0.05), other parents (*p* < 0.01), and teachers in child-care facilities (*p* < 0.01) are significantly less important as contact partners concerning AOM treatment in their child. Regarding the “wait-and-see” strategy, “several-child parents” agree considerably more often to wait and consult the doctor again, when symptoms worsen (*p* < 0.05) before giving an antibiotic. However, in comparison to the “single-child parents” they would rather immediately give an antibiotic (*p* < 0.01) when severe symptoms are present right from the beginning. Regarding their requests for medical prescriptions for their child with AOM they do not differ from “single-child parents”.

## Discussion

The aim of our study is to elicit parental knowledge/beliefs, attitudes, and experiences on AOM and its treatment in the German health care context. In general, the results provide first insights on how parents might think about AOM and experience AOM and its treatment within the German health care system. With respect to knowledge/beliefs about AOM, parental answers indicate a realistic view of key symptoms but show uncertainties regarding underlying causes and the natural course of the disease. Knowledge about antibiotics reveals misconceptions regarding effectiveness in AOM treatment and a more realistic view on undesired effects. Around 40 % of all parents are generally willing to follow a “wait-and-see” strategy, but for severe symptoms, around the same portion generally prefers the immediate use of an antibiotic. Experiences with AOM therapy show that parental request rates for antibiotic treatment strongly differ from the reported rates of actual prescription, indicating that antibiotics are around three times more likely to be prescribed for children with AOM than expected by the parents.

### Parental knowledge/beliefs about AOM, treatment of earache and effects of antibiotics

The present results indicate that parents seem to have a fairly realistic view of key symptoms of the disease, as the 92.7 % generally agree that earache is associated with AOM and 54.3 % generally agree that fever is part of AOM. Uncertainties exist concerning causes and the natural course of the disease. Sixty-six percent of the parents generally agree that bacteria cause AOM, whereas 20.1 % generally agree that viruses cause AOM. 30.5 % do not generally agree that viruses cause AOM. A relatively high proportion states not to knowing the cause (11.6 % for bacteria, 15.9 % for viruses), and 4.3 and 10.9 %, respectively, do not answer. Thus, the fact that viruses are mostly involved in the pathophysiological mechanisms of AOM [46, 47] does not seem to be widely known. AOM-experienced parents significantly less often believe viruses to be involved than non-AOM-experienced parents. Given the fact that parents know well that antibiotics are effective against bacteria [48, 49], these findings might reflect the experiences of 70.2 % of the AOM-experienced parents in our sample that their child with AOM has previously been treated with an antibiotic. The reported proportion of antibiotic prescriptions in our sample largely corresponds to findings of other authors [50, 51].

Special emphasis should be placed on the parental perception towards the natural course of AOM and the need for antibiotics. Only 8 % of the respondents in the present survey generally agree to the statement that AOM resolves spontaneously, whereas 53.6 % do not generally agree. These opinions are supported by the view of 45.7 and 42.8 % of the parents, who, respectively, generally and partly agree that AOM needs antibiotic treatment, which indicates that parents might considerably underestimate the self-limiting character of uncomplicated AOM in children. This assumption is also supported by a previous survey reporting a high proportion of parents who believe antibiotics are necessary when treating AOM (85 % for Finland, 55 % for The Netherlands) [52].

Most of the parents in our sample generally (45.7 %) or partly (42.8 %) agree that AOM needs antibiotic treatment and although 71 % generally agree that analgesic/antipyretic drugs are the best treatment for earache in AOM, 52.1 % generally agree antibiotics are the best pain-relieving therapy in earache. The latter is in accordance to results from other authors [49, 53] and may be explained by the finding that 56.6 % of the parents in our sample generally agree to the statement that antibiotics lead to rapid pain relief. A fast pain relief is of great importance to parents, since AOM in children gives rise to considerable burdens for the affected children as well as for their families [51, 54]. However, the expected rapid analgesic effect (within 24 h) in the course of AOM treatment in children is not confirmed by a recently published Cochrane analysis [20]. This review shows that, compared to placebo, antibiotics do not lead to a significant pain reduction within 24 h. The review also demonstrated that, compared to a placebo or watchful waiting (“wait-and-see”), antibiotics do not reduce severe complications or recurrence rate of AOM. The majority of the respondents in the present study is aware of possible harms associated with antibiotic treatment such as negative effects on the gastrointestinal tract, which actually is seen in one of every 14 antibiotic-treated children [20], or possible inefficiency after frequent use (antimicrobial resistance), which is known to be an increasingly national and international threat for general public health [19, 55]. The high parental awareness towards the increased risk of antimicrobial resistance associated with antibiotic overuse demonstrated in the present study is consistent with results of other investigations [49, 52, 56].

In summary, parental knowledge and beliefs concerning AOM and its treatment and the effects of antibiotics turn out to be heterogeneous. This might be due to miscommunication between parents and physicians. Whatever the reason, these results could serve as a basis for developing patient-centered and evidence-based information on the treatment of AOM for parents.

### Parental attitudes towards AOM treatment in their own child

Our study shows that the pediatrician is the most important contact partner for parents who seek medical advice regarding the treatment of AOM. This finding is consistent with sickness fund data from Germany on the utilization of pediatricians or family doctors with sick children up to the age of seven [57]. However, our study shows two opposing trends concerning parental attitudes towards the two different AOM therapeutic concepts available for treating their child, either allowing a 2-day observational period before giving an antibiotic or preferring immediate antibiotic treatment. While almost 40 % would generally accept the “wait-and-see” strategy and only give an antibiotic once symptoms have persisted for 2 days or worsened overnight, about the same percentage would not generally accept this strategy. For severe symptoms, 44.2 % would immediately administer an antibiotic, whereby, compared to “single-child parents”, “several-child parents” prefer this concept (*p* < 0.01). These results indicate that around 40 % of the parents might generally favor the “wait-and-see” strategy, but might prefer an immediate antibiotic use if the child is suffering greatly. This finding suggests that many parents take antibiotics as the most effective therapy compared to other options. The relatively high proportion of parents rejecting initial observation corresponds to the results found by Finkelstein et al. [32], who conducted a survey dealing with physicians’ use of initial observation in AOM and the parental acceptance of this strategy. In contrast to our results, the investigators additionally identified an association between educational level and parental acceptance of initial observation. It should be noted that parental acceptance of initial observation can be supported when the doctor gives a brief explanation for this strategy [58]. Adherence is also enhanced when parents are instructed to seek follow-up care if the symptoms persist without receiving an additional prescription for antibiotics (with the advice to hand it in, if symptoms fail to improve) compared to not receiving a prescription [59].

### Parental experiences with AOM treatment

International guidelines [22, 60, 61] and recently published national overviews [47, 62] recommend that children aged 2 years and older with uncomplicated unilateral AOM receive initial observation including symptomatic treatment with an analgesic drug as first line therapy. An antibiotic should be added if symptoms fail to improve within 48 to 72 h. The use of other agents, such as naturopathic remedies, eardrops with a pain-relieving substance, or nasal drops with decongestant, is not explicitly recommended.

With respect to parental requests for medical treatment and the reported actual prescriptions, we find two trends: (1) For analgesic/antipyretic drugs, nasal drops with decongestant, naturopathic remedies, and pain-relieving eardrops, the rates of parental requests and actual prescriptions are in high concordance. (2) For antibiotics, there is a striking discrepancy between reported parental request rates and reported prescribing rates, indicating that antibiotics might be around three times more likely to be prescribed for children with AOM than expected by the parents. This finding contradicts the frequently expressed view that parents often put pressure on doctors to prescribe an antibiotic for their child with AOM [55]. We cannot fully exclude the possibility that there might be a recall bias especially concerning requests for antibiotics and actual antibiotic prescriptions, which is much larger than the difference found for all other treatment options. Nevertheless, in accordance with our results, other studies have also suggested that antibiotic overuse is not caused by parental pressure [49, 63]. Based on the present results, possible reasons for the marked difference between parental request rates and doctors’ prescription rates in our sample remain unclear. The relatively low parental tendency to ask for an antibiotic might have several reasons, such as having concerns about antibiotic overuse or antibiotic resistance [52, 64] or a tendency not to ask doctors for a special medication, e.g., because parents trust them and rely on their decisions [65].

From the physicians’ perspective, the question arises as to why actual antibiotic prescription rates in the present study are as high as reported. It could be concluded that the “wait-and-see” strategy is applied less than could be expected based on the guideline recommendations. Determinants other than objective medical criteria, such as perceived parental expectation might play a role in antibiotic prescription [29, 63, 66]. Another reason for overuse of antibiotics may be diagnostic uncertainty resulting in over-diagnosis of AOM [27, 29, 67–69]. As Täthinen et al. suggest, treatment practices and parental expectations seem to interact [52]. Therefore to achieve a change in treatment practices, both parental views and physicians’ attitudes and practices have to be addressed.

### Parental experience of AOM and children’s health

Our analysis demonstrates that prior experience of AOM does not influence parental knowledge and attitudes, except that AOM-experienced parents are less inclined to agree that AOM is caused by viruses. However, having two or more children (implying more general experience of children’s health) is associated with significant differences compared to having a single child: “Several-child parents” regard nasal drops with decongestant and naturopathic drugs more often as being the best therapy for earache. Although they classify antibiotics less often as best pain treatment, they prefer more often immediate antibiotic use in cases of severe symptoms. Thus, general experience of children’s health might have a stronger influence on parental knowledge about and attitudes to AOM and its therapy than concrete experience with AOM. This conclusion is in accordance with results from research by Kuzujanakis et al. [30], who found a significant association between having more than one child and correct parental antibiotic knowledge. However, in contrast, a recently published study that investigated parental views on childhood fever found no correlation between having more than one child and knowledge about antibiotic effectiveness on bacterial infections [70], whereas personal parents’ experience with serious illness in the own child was significantly associated with more concerns about health problems related to fever. In our sample higher parental educational level is not associated with higher antibiotic knowledge. This result is in contrast to other studies that see a correlation between parental education degree and the parental antibiotic knowledge [30, 71], the tendency to expect or ask for an antibiotic [30, 72] the frequency of antibiotic prescriptions [29], and the use of antibiotics with their child [30, 63]. The present data allow no clear conclusions to be drawn on the reasons for this discrepancy.

The current findings may be of considerable relevance for several reasons: (1) The results may provide a basis for a better understanding of parental views on AOM and its therapy. To confirm these findings, further investigations with a representative parent sample of parents in Germany are called for. (2) The results give some indication of parental concepts and misconceptions on AOM and its therapy. Since decisions on treatment options may be influenced by both physicians *and* parents, the findings may serve as a basis for developing evidence-based information for parents to support parental health literacy and for fostering shared decision-making processes between parents and physicians. (3) The reported prescription rates of antibiotics may lead to the assumption that actual guidelines on AOM management in children, especially the option of “wait-and-see”, may be used less often than could be expected. Further investigations are needed to elucidate this hypothesis.

### Strengths and limitations

The main strength of this study is that it represents a first survey in Germany that investigates knowledge/beliefs, attitudes, and experiences towards AOM and its therapy in parents with children aged 2 to 7 years. While not claiming to be representative, the results provide initial insights in parental views on AOM and its treatment. Although the results do not allow generalization so far, they still might serve as a starting point for further investigations of the German population. Limitations of the survey include the small number of respondents, a low response rate and the convenience sampling strategy applied for recruiting childcare facilities. Most of the participants are female and are not single parents, and more than half of them hold a university degree. Therefore, a possible selection bias cannot be excluded. On the other hand, this selection may reflect–at least in part–an approach to a realistic depiction of the group of parents who usually deal more often with childcare. The survey may also be subject to a non-response bias, because it only includes those childcare facilities and those parents that voluntarily agreed to participate and we had no means of analyzing the non-responders. Additionally, due to the lack of data concerning the time period between the last experienced AOM and answering the questionnaire, there might be a recall bias arising from a possible time delay between experiencing the acute disease and giving statements from memory. This time delay might bias the actual experiences and expectations and influence the answers.

## Conclusions

We present the results of an exploratory survey in the German health care system that investigates knowledge/beliefs, attitudes, and experiences towards AOM and its therapy in parents with children aged 2 to 7 years. Parental knowledge and beliefs on AOM and its therapy reveal uncertainties especially with respect to underlying causes and the natural course of the disease as well as misconceptions concerning antibiotic effects in AOM, indicating that there is a need for more evidence-based information that improves parents’ health literacy and enhances SDM in the treatment of children with AOM. Results on experiences with AOM therapy show that parental request rates for non-antibiotic options are in line with actual prescription rates, while antibiotics are three times less often requested by the parents than actually prescribed. This finding contradicts the hypothesis that parents put pressure on doctors to prescribe an antibiotic for their child with AOM. Further investigations are needed to clarify these findings.

## Additional file

Additional file 1:
**Questionnaire (English translation).**

## Abbreviations

AOM: Acute otitis media
ENT specialist: Ear-nose-throat specialist
HI: Health insurance
SDM: Shared decision making
